# Women’s views about contraception requirements for biomedical research participation

**DOI:** 10.1371/journal.pone.0216332

**Published:** 2019-05-08

**Authors:** Kristen A. Sullivan, Margaret Olivia Little, Nora E. Rosenberg, Chifundo Zimba, Elana Jaffe, Sappho Gilbert, Jenell S. Coleman, Irving Hoffman, Tiwonge Mtande, Jean Anderson, Marielle S. Gross, Lisa Rahangdale, Ruth Faden, Anne Drapkin Lyerly

**Affiliations:** 1 Center for Bioethics and Department of Social Medicine, University of North Carolina School of Medicine, University of North Carolina at Chapel Hill, Chapel Hill, North Carolina, United States of America; 2 Kennedy Institute for Ethics, Georgetown University, Washington, D.C., United States of America; 3 Department of Health Behavior, University of North Carolina Gillings School of Global Public Health, University of North Carolina at Chapel Hill, Chapel Hill, North Carolina, United States of America; 4 UNC Project Malawi, Lilongwe, Malawi; 5 Berman Institute of Bioethics, Johns Hopkins University, Baltimore, Maryland, United States of America; 6 Division of Gynecology and Obstetrics, Johns Hopkins University School of Medicine, Johns Hopkins University, Baltimore, Maryland, United States of America; 7 Institute for Global Health and Infectious Diseases, University of North Carolina School of Medicine, University of North Carolina at Chapel Hill, Chapel Hill, North Carolina, United States of America; 8 Department of Obstetrics and Gynecology, University of North Carolina School of Medicine, University of North Carolina at Chapel Hill, Chapel Hill, North Carolina, United States of America; University Medical Center Utrecht, NETHERLANDS

## Abstract

The scientific and ethical importance of including women of reproductive age in biomedical research is widely acknowledged. Concerns about preventing fetal exposure to research interventions have motivated requirements for contraception among reproductive aged women in biomedical studies–often irrespective of risks and benefits or a woman’s actual potential for pregnancy, raising important questions about when such requirements are appropriate. The perspectives of women themselves on these issues are largely unexplored. We conducted 140 interviews, 70 in the U.S. and 70 in Malawi, with women either living with or at-risk for HIV, exploring their views about the practice of requiring contraception in clinical trials. A majority of women interviewed from both countries indicated overall support for the practice, with seven themes characterizing advantages and disadvantages raised: reproductive control, health effects, prevention of fetal harm, burden on women, deferral to authority, autonomy regarding enrollment and birth control method, and relationship concerns. While women in the US frequently raised prevention of fetal harm as a key advantage, many other positives noted by women in both countries were related to contraception use in general, not specific to a trial context. With regard to disadvantages, U.S. women tended to focus on *biomedical* risks such as side effects and impact on fertility, whereas Malawian women focused on the *social* risks of contraception requirements, including violations of trust in marital relations and suspicions of potential infidelity. Given the potential benefits and burdens highlighted, contraception in research should be sensitive to actual fetal risk assessments; directed where justified at optimizing effective pregnancy prevention; responsive to women’s reproductive preferences; and made available as an ancillary benefit even where risk thresholds do not justify requirement–in order to facilitate trials that are both ethical and robustly oriented around the interests and lives of women who will participate in them.

## Introduction

The scientific and ethical importance of including women of reproductive age in biomedical research is now widely acknowledged. Sex-based differences in disease mechanisms, treatments and interventions require inclusion of women to generate evidence for the care of female patients [1–3]. However, concerns regarding potential harms to fetuses of women who are or may become pregnant during the course of a trial have historically restricted women’s participation across biomedical research. While such restrictions are justified in some circumstances, they are often applied without robust consideration of maternal and fetal risks and benefits or consequences of exclusion. As a result, there are significant gaps in the evidence base for women’s health, and individual women may be unjustly denied the prospect of direct benefit from participation in some studies [4,5]. Though the inclusion of women and their interests in biomedical research has increased in recent years, robust inclusion remains elusive, as marked by enduring concerns about justice and fair access to benefits of investments in advancing biomedical knowledge [6]. Additionally, fetal protection concerns have also motivated contraception requirements among biomedical studies that do enroll reproductive-aged women, raising important questions about the appropriateness of these widespread requirements.

One pressing question is when, and under what conditions, women of reproductive potential should be required to use contraception as a precondition for enrollment in biomedical research. On the one hand, requirements for effective contraception have been considered an important way to prevent fetal exposure in biomedical research [7,8] and ensure women’s access to research where study interventions have the potential to cause fetal harm [9]. On the other hand, requirements for contraceptive use often persist independently of a woman’s actual potential for pregnancy, or in the absence of a meaningful assessment of the risks and benefits [10]. Further, provisions in U.S. Food and Drug Administration (FDA) guidance for the conduct of research with women of reproductive potential allow deferral of certain reproductive toxicity studies if requirements for contraception are instituted [8]; this raises the possibility that contraception requirements may at times be less about reducing theoretical fetal risks, and more about minimizing research and development (R&D) timelines and costs. These issues have raised concerns about whether current patterns of contraceptive requirements disrespect women’s autonomy, or unduly impose the risks and burdens of contraceptives to research participants [11,12], and unfairly impede access to research carrying the prospect of direct benefit. Additionally, concerns have been raised that the widespread use of hormonal contraception in biomedical research has problematically restricted our understanding of variations in pharmacokinetics associated with normal or natural menstrual cycles occurring in the absence of synthetic hormones [10].

In order to address these important concerns, improved guidance regarding contraception requirements in biomedical research is needed. There are currently no harmonized international regulatory guidelines for such requirements [13]. The U.S. FDA has recommended that “women be counseled about reliable use of contraception or abstinence … [but does not] specify the type of contraception used because FDA believes decisions of that nature are best left to the woman in consultation with her health care provider” [7]. U.S. regulations governing research with human subjects are silent on the use of contraception [14], and the Council for International Organizations of Medical Sciences (CIOMS) *Ethical Guidelines for Research* emphasizes the importance of access to contraception for women of childbearing potential in clinical studies, but does not address the ethical complexities of contraception requirements [9]. Individual Institutional Review Boards (IRBs) generally develop institution-specific guidance, which can vary widely. Additionally, limited suggested guidance has appeared in the academic literature; the two proposals that have been published used the FDA’s now-retired, letter-based labeling categories, and differ on several key points, including when, if ever, investigators may require abstinence or the use of contraception as an inclusion criterion [11,15].

It is important that researchers and policy makers take account of the diverse array of perspectives of women as they shape evolving regulations and best practices to address pregnancy, fertility, and the role of contraception in clinical trials [16]. In recent years, research subjects’ views have been recognized as a critical component to adequate research ethics analysis and oversight [17]. Although potential participants may not be knowledgeable about historical, institutional, or other factors that bear on policy options, they may well be aware of other morally relevant considerations that policy makers and scholars might otherwise overlook, including the ways in which their own values and life context affect how they view clinical trials when contraception is required.

Yet women’s views are largely missing from the discussion about contraception in research. We could identify no studies that asked women their views about the advantages and disadvantages of contraception requirements in research contexts, and only two studies of trial participants’ opinions that touched on the subject. A qualitative study in Uganda explored male and female participants’ understanding of the informed consent process for required contraceptives and reasons for noncompliance with contraception requirements in trials. While all respondents described being advised to avoid pregnancy during trial participation, factors reported to be associated with pregnancy included the belief that the investigational product presented low fetal risk, a need for children, male partner unwillingess to use condoms, and side effects of contraception [18]. A small survey of US women queried willingness to participate in hypothetical trials of drugs to reduce breast cancer risk, and found that requirements for the use of specific contraceptives, as opposed to women’s own choice of birth control method, reduced women’s willingness to participate [19]. Thus, debates among IRB members, researchers, regulators and policymakers have proceeded in the face of little empirical information about the views and concerns of those potentially most affected by such policies.

To address this void, we elicited women’s views on contraception requirements as part of a larger qualitative study conducted by the Pregnancy and HIV/AIDS: Seeking Equitable Study (PHASES) project [20]. Our study interviewed pregnant and recently pregnant women living with or at risk of HIV/AIDS, exploring their views about and experiences around clinical trial participation. The context of HIV/AIDS research is particularly relevant to the exploration of contraception requirements in clinical trials for three reasons. First, considerable research has been conducted with women living with or at risk for HIV/AIDS, and issues related to pregnancy and contraception have been a matter of debate [21–23]. Second, women living with or at risk for HIV/AIDS are disproportionately low-income and face a range of challenges regarding access and consent to research participation in health care settings; understanding particular challenges of low- and middle- income country (LMIC) contexts where HIV research is often conducted and to which findings will be most relevant is critical [18,24–26]. Third, given the international partnerships that characterize HIV/AIDS research, standard research practices need to be sensitive to implications they may have for critical research in international settings. We therefore conducted a study of pregnant women in both the US and in Malawi, a country with a high prevalence of HIV/AIDS and a robust research infrastructure.

## Methods

The data for this analysis were collected as part of a larger project, PHASES, which aims to develop guidance for ethically acceptable research on HIV treatment and prevention during pregnancy. We conducted qualitative, in-depth interviews using a semi-structured guide, as described previously [20]. The data for this analysis, collected between August 2016 and April 2017, pertain to women’s opinions about common contraception requirements for women participating in clinical trials. Interviewers described the common practice in clinical trials of requiring women to use birth control in order to participate, highlighting that two forms of birth control are often mandated, and that male partner vasectomy may not count as a type of birth control [12,27]. If the woman does not agree to use birth control, then she cannot participate in the study, even if she does not currently have a sexual partner (see Box 1). The purpose of this approach was to surface participants’ initial reactions to this widespread requirement in biomedical research with women of reproductive potential, including their thoughts around when it would and wouldn’t be appropriate. Therefore, no potential justifications for the contraception requirement were provided to the participants. Interviewers asked women to discuss what they thought about this practice of requiring contraception with open-ended probing of advantages and disadvantages, asking them to reflect on requiring one versus two forms, as well as their overall opinion.

**Box 1. Contraception requirement language used in interviews**

“In some research studies, if a woman who is not pregnant wants to participate she must agree to use birth control (for example condoms, birth control pills, an IUD, an implant, or previously having her tubes tied). Many times she will be asked to use two forms of birth control (for example a condom plus an IUD) for extra protection. It may not count if her partner has had a vasectomy. If she does not agree to use birth control then she cannot participate in the study, even if she does not currently have a sexual partner.”

Written, informed consent was completed prior to the interview. Interviews in Malawi were conducted in Chichewa by one of two researchers who are native speakers; all U.S. interviews were conducted in English. Demographic questions were also completed. The research was approved by the Institutional Review Boards at the University of North Carolina at Chapel Hill and Johns Hopkins Bloomberg School of Public Health and the National Health Science Research Committee of Malawi.

### Participants

One hundred and forty interviews were conducted with pregnant or recently pregnant women living with or at risk for HIV; 70 at sites affiliated with UNC Project Malawi in Lilongwe, Malawi, and 70 at U.S. clinics affiliated with the University of North Carolina in Chapel Hill, North Carolina or Johns Hopkins University in Baltimore, Maryland.

Eligibility criteria for participation included that the adult woman either be pregnant or recently pregnant (within 2 years). Nearly half of the Malawi sample (n = 33) were purposively selected for their previous participation or attempted enrollment in a HIV prevention or treatment study during pregnancy, all of which had some form of contraception requirements; these participants did not have to meet the eligibility criteria of currently being pregnant or recently pregnant. Reflecting the broader purposes of the PHASES project, the sample equally represented women living with HIV and those at-risk. At both study sites in the US, women at-risk for HIV were receiving health care and were recruited from obstetrics clinics in jurisdictions with elevated incidence of HIV or AIDS among adolescents and women aged 15 to 45 years.

### Analysis

The analytic approach was informed by thematic analysis [28], and described in detail elsewhere [20]. Interviews were recorded, transcribed verbatim, and translated as necessary. Coding was an iterative process using NVivo software, with initial codes developed a priori from the interview guide. As analysis progressed, new codes were added as needed, and data was re-coded as applicable. Emergent themes were identified from making comparisons within and across individuals utilizing data display matrices, and identifying thematically and conceptually related categories. These themes were: reproductive control, health effects, preventing fetal harm, burden on women, deferral to authority, autonomy to enroll and choice of birth control method, and relationship concerns. Quotations representing each of the themes were selected and are attributed by pseudonym, country of residence, age, HIV status, and race/ethnicity.

## Results

### Participant characteristics

Participant demographics are presented in Table 1. Participant age ranged from 18 to 50 (mean age Malawi = 29.4, U.S. = 29.8), with the largest proportion of women in both countries in the 25–34 year age group. Seventy percent (n = 49) of U.S. participants were Black/African-American, and all women in Malawi were black Africans. Twenty-three percent of U.S. women were college graduates or higher, and 20% of participants in Malawi completed secondary school or higher. Thirty-six percent of U.S. women and 90% of Malawian women were married. It is worth noting that the concept of marriage in Malawi is colloquially considered to be broader than the explicit legal definition, and includes cohabitating couples who typically raise children together. In both countries, the majority of participants identified as Christians (67% in the US, 89% in Malawi), and 50% of participants were HIV-positive (35 in U.S. and 35 in Malawi).

**Table 1:** Demographic characteristics of women participating in PHASES.

Characteristic		U.S. (*n* = 70)		Malawi (*n* = 70)		TOTAL (*n* = 140)	
		*n*	%	*n*	%	n	%
Age							
	18–24	18	26%	17	24%	35	25%
	25–34	31	44%	39	56%	70	50%
	35–44	20	29%	12	17%	32	23%
	45+	1	1%	2	3%	3	2%
Race/Ethnicity							
	Black/African American/Not Hispanic	49	70%	70	100%	119	85%
	White/Not Hispanic	12	17%	—	—	12	9%
	Hispanic/Latina	7	10%	—	—	7	5%
	Asian/Other	2	3%	—	—	2	1%
Born outside the US		9	13%	70	100%	79	56%
Education							
	U.S.:						
	Some HS	12	17%				
	HS grad/GED	19	27%				
	Some college/Assoc degree	23	33%				
	College graduate	6	9%				
	Post graduate degree	10	14%				
	Malawi:						
	None			9	13%		
	Primary: Some to completed			32	46%		
	Some secondary			15	21%		
	Completed secondary			11	16%		
	Post-secondary			3	4%		
Marital status							
	Single	24	34%	3	4%	27	19%
	Married	25	36%	63	90%	88	63%
	Living with partner	19	27%	—	—	19	14%
	Divorced or separated	2	3%	3	4%	5	4%
	Widowed	—	—	1	1%	1	0%
Number of pregnancies							
	1	17	24%	7	10%	24	17%
	2–3	26	37%	30	43%	56	40%
	4+	27	39%	33	47%	60	43%
HIV positive		35	50%	35	50%	70	50%
Religion							
	Christian						
	Catholic	8	11%	14	20%	22	16%
	Protestant	39	56%	48	69%	87	62%
	Jewish	—	—	1	—	1	0%
	Muslim	—	—	6	9%	6	4%
	None	21	30%	—	—	21	15%
	Other/Not reported	2	3%	1	1%	3	2%

More than half of women in both the U.S. (60%) and Malawi (58%) indicated overall support for the practice of requiring contraception in clinical trials; about a fifth opposed the requirement, and the remainder–also about a fifth–were unsure or said it depends on the circumstances (see Table 2). Those who were unsure or said it depends often believed the requirements should be determined based on the potential risks of exposure to the study drug if pregnancy were to occur during the course of the trial. Others felt a requirement for one type of birth control was reasonable, whereas requiring two forms placed an unnecessary burden on the woman and/or couple. In addition to their overall assessment, participants discussed what they saw as advantages and disadvantages of the practice. Their responses diverged both within and across countries, with several emergent themes being specific to one country or the other, as described below and summarized in Table 3. Many women, regardless of overall support for or opposition to a contraceptive requirement, articulated nuanced and multifaceted opinions, including the different contexts in which and reasons why they felt it to be appropriate or not.

**Table 2:** Participants’ overall views by country on contraception requirements for clinical trial participation.

Contraception Requirements Opinion	U.S. (*n* = 70)		Malawi (*n* = 70)		Total (*n* = 140)	
	*n*	%	*n*	%	*n*	%
Support	42	60%	38	56%	80	58%
Oppose	15	21%	12	18%	27	20%
Unsure/It depends	13	19%	18	26%	31	22%

**Table 3:** Themes emergent across participants’ assessments of advantages and disadvantages of contraception requirements.

Domain	Advantages of a requirement		Country	Disadvantages of a requirement		Country
Reproductive control	Access to birth control/prevention of unintended pregnancies		U.S. & Malawi			
	Pregnancy prevention enhanced by 2 types of birth control		U.S.	Reduced control of pregnancy timing		U.S. & Malawi
	Male partners respect requirement so women get access to benefits		Malawi			
Health effects	Protection from sexually transmitted diseases (with condom use).		U.S	Side effects of birth control		U.S. & Malawi
	Improved birth spacing		Malawi	Side effects of birth control magnified with two forms of contraception		U.S.
Preventing fetal harm	Fetal protection from unknown risks of study drug		U.S.			
	Requirement is appropriate when potential risk for fetal harm is present*		U.S	Requirement is not appropriate when potential risk for fetal harm is absent*		U.S.
	Liability protection for researchers		U.S.			
Burden on women				Birth control is unnecessary if not sexually active		U.S. & Malawi
	Low incremental burden on women already using one form		U.S.	Using two types of birth control is burdensome.		U.S.
					- One long-acting/highly efficacious should be sufficient	
				Desire to minimize medication use and be “natural”		U.S.
Deferral to authority	Researchers have the right to establish the rules		U.S. & Malawi	**—**		
Autonomy to enroll and choice of birth control method	Women can make informed choice about participation in the study		U.S.	**—**		
	Participants get to select which form(s) of birth control they use		U.S. & Malawi			
Relationship concerns	—			Partner may suspect infidelity if he is away and woman is using contraception, or he has had vasectomy		Malawi
				Man may find an alternative sexual partner to avoid using condoms		Malawi

*Participants indicated appropriateness of requirement is dependent on potential of risk for fetal harm

### Reasons supporting clinical trial contraception requirements

Women cited varied reasons for supporting contraception requirements, including increased reproductive control, health benefits, preventing fetal harm, a low perceived burden on the woman, the authority of the researchers to establish the rules, and that women are free to decide if they want to participate or not and to choose the form(s) of contraception they prefer.

#### Increased reproductive control

Respondents commonly viewed contraception requirements as offering a benefit to participants by providing access to and support for birth control use, and therefore, protection from unintended or unwanted pregnancies. In many cases, this was expressed as general support for contraception use, not necessarily limited to the context of trial participation.

*“(I think it is a) good rule, because … getting pregnant is a life changing situation. It could alter your life in so many ways. It has altered mine. And … it opens up a whole another can of worms. So if you’re using a contraceptive… to help prevent [pregnancy], yes. Because right now*, *you just—when the intimacy come, the fun of having sex comes, you’re not thinking about pregnancy. . . .”*

Jayla, U.S., 37 years, HIV negative, African American/Black

“*I think the rule is good because sometimes you can say I am tired of being alone*, *but you do not have a husband and so you find a boyfriend… but you are not getting an injection or anything else*. *So once you have sex with someone*, *you fall pregnant immediately*. *So when you accept this rule*, *you avoid a lot and you do not get pregnant*.*”*

Mary, Malawi, 26 years, HIV negative, Black African

Some U.S. participants also viewed the two forms of contraception as enhancing protection against pregnancy.

*(E)specially for younger girls… Mainly because one birth control sometimes fails*, *like if someone is on the pill*. *I was on the pill and didn’t use condoms*. *I ended up pregnant when I was 16*, *so I would consider that* …*”*

Veronica, U.S., 31 years, HIV negative, white

Particularly in Malawi, where men commonly are the primary decision-makers around birth control use, some participants saw requiring contraception as promoting women’s interests. One woman described how, if the husband has given consent for his wife to participate in the study, the birth control requirement would grant her an opportunity to defer further childbearing that she might desire but otherwise not have outside the context of the study.

“*Since they have put family planning that is why it is good*, *because if you have agreed that you will join the study with your husband*, *and there are husbands who don’t want contraceptives…*. *you get opportunity of resting*, *so go and get contraceptives*.*”*

Violet, Malawi, 31 years, HIV negative, Black African

#### Health benefits

Participants in the US also commonly noted the benefit of two forms of birth control offering protection against sexually transmitted infections, especially when one form is a condom. These reasons were also not typically limited to the research context.

*“(I)t’s more protection. Because one form will prevent you from getting pregnant, and another one will prevent you from getting any STD. Like if it’s a condom, it will prevent you from STD*. *If it’s the pill, it will only prevent you from getting pregnant.”*

Andrea, U.S., 32 years, HIV negative, Latina

Women in Malawi discussed that provision of contraception under such trial requirements had potential health benefits to women and their offspring resulting from desired birth spacing.

“*(L)et’s say you have a husband in the house*, *it means when you are using family planning you cannot get pregnant fast*. *Meaning you can have 2 or 3 years while your body is still fine and you are not pregnant*. *You also give a chance for the child who is little to grow well*. *That is the advantage*.*”*

Ellen, Malawi, 26 years, HIV positive, Black African

#### Preventing fetal harm

Protection from fetal harm was a reason in support of contraception requirements in trials that commonly emerged unprompted from women in the US.

“*Because I feel like they’re [researchers] looking out for the interest—obviously whatever they’re testing won’t be good for the baby*. *So if she knows that … she doesn’t want to have any kid*, *or not get pregnant within a certain timeframe*, *then I think it’s a good rule*.*”*

Tiana, U.S., 36 years, HIV negative, Black/African American

Notably, only one participant in Malawi described preventing fetal harm as a benefit of the requirement.

*“(I)f you get pregnant you can stop taking the medication… because it might cause certain problems for the baby… So it’s good not to get pregnant (participants should) use a family planning method*, *even tubal ligation…”*

Leoni, Malawi, 34 years, HIV negative, Black African

Some participants in the US believed contraception requirements should be sensitive to the possibility of risk to potential offspring, and are appropriate for studies where such risk is present, but should not be a blanket requirement.

“*Again*, *it all depends on what the study is and how it could affect a fetus if you were to get pregnant or not if you could harm it*, *kill it*, *whatever*. *It depends on the scenario and the type of study it is… If it’s one that’s potentially able to harm the fetus then*, *yes*, *it’s a good rule*.*”*

Dominique, U.S., 34 years, HIV positive, white

A participant noted the desire of researchers to protect the pregnancy and the fetus, and also guard against liability for possibly causing harm.

“*I think that that is important because some medical interventions … could be potentially harmful for the pregnancy*, *and I think*, *too*, *the researchers want to protect the patient but they also want to protect the liability for themselves and wouldn't want to expose the patient to any sort of toxins to the pregnancy or any kind of medication that could be potentially harmful for the fetus*.*”*

Jasmine, U.S., 32 years, HIV negative, multi-racial

#### Low incremental burden for some women

Several women in the U.S. noted that a contraception requirement would not be a significant additional burden, as long as they already are using birth control.

“*I think if I was at a time in my life where I wasn't going to have kids at that point or I wasn't planning on getting pregnant*, *then I don't see how that rule would really impact it*. *I mean I would take birth control anyway*, *so for me it wouldn't impact me one way or the next*.*”*

Sarah, U.S., 32 years, HIV negative, white

“*That's not too bad…because if you get two forms like a condom and a shot or a condom and a pill then that's not bad at all*, *because even without the study you'd probably be taking that Depo shot or you're probably using those condoms… I would agree with that*.*”*

Jodi, U.S., 26 years, HIV positive, African-American/Black

#### Deferral to authority

Some women in both the US and Malawi noted what they perceived as the legitimate authority of the researchers to develop their own rules for participants in their studies and that the rules must be followed by participants.

*“I feel like that's a good rule—because, you can't make the rules up yourself*. *If you want to be a part of the study, you have to follow the rules that they come up with. So if they say that you have to do this a certain way, made you do two…, then–that's what you need to do.”*

Ayana, U.S., 22 years, HIV negative, African-American

“*(Y)ou just have to follow the rules*. *Because the rule has been put in place*, *you have to follow it*. *Whether your husband had vasectomy*, *you still have to use birth control…”*

Fredah, Malawi, 25 years, HIV negative, Black African

#### Autonomy to enroll and choice of birth control method

Numerous respondents supported the practice of requiring contraception because participation in studies is optional, and potential participants would be informed of such a requirement, provided a choice of birth control method(s), and could decide to enroll or not.

*“(A)s long as she’s educated on it … If it’s just too much, and that’s not a risk she wants to take, then she can always just say, ‘No, I can’t do the study.’ As I said*, *as long as she goes into it with eyes wide open, has all the options available, and can switch if there’s any problems, then I don’t see a problem with it.”*

Sabrina, U.S., 33, HIV positive, African-American/Black

“*It is good because you accepted on your own that you will use birth control methods until the research ends*. *No one forced you*, *you accepted on your own*.*”*

Yvonne, Malawi, 37 years, HIV positive, Black African

“*This rule does not cause me any problems…*. *(Y)ou can just choose what you use and which method suits you*.*”*

Fredah, Malawi, 25 years, HIV negative, Black African

### Reasons for opposing clinical trial contraception requirements

Women also articulated varied reasons in opposition to contraception requirements, including reduced reproductive control, negative health effects of birth control, the undue burden compliance places on the woman, and in Malawi, concerns around the requirement straining the relationship with their partner.

#### Reduced reproductive control

A common theme emerging from opposition to birth control requirements was the perceived effect of giving the researchers control over when a woman can get pregnant.

“*It’s not good because it requires you to use contraceptives even if you don’t want to*. *So it might be like they are forcing you to do family planning while you don’t want*.*”*

Violet, Malawi, 20 years, HIV negative, Black African

“*…I wouldn't want nobody to tell me when*, *how I can get pregnant*. *Things change*, *you know*, *every day*. *You might don't want a child today but you might meet the love of your life and he might want a child in the next six months*, *you know*. *So I would not want nobody to determine when I can start my family or expand my family*, *so for me*, *no*, *I would not agree to join something that tells me when I can have my child*.*”*

Felicia US, 37 years old, HIV negative, African American/Black

#### Negative health effects

Another central concern was the potential side effects of birth control, and the recognition that these effects vary between individuals, making it a more challenging requirement for some.

“*(E)veryone’s body reacts different to birth control*, *so the effects of the birth control may make it uncomfortable for the person*.*”*

Chanise, U.S., 23 years, HIV negative, African American/Black

“*There are some women who respond very badly to contraceptives*. *It may cause certain problems for them*. *So they are the people that need to be considered properly or exempted*.*”*

Saliza, Malawi, 34 years, HIV positive, Black African

Some women in the US expressed worry that taking two forms of birth control may magnify the risk of side effects, including potentially reducing women’s long-term fertility, especially over a multi-year study.

“*(I)t also can be very harmful to your body*, *so if you eventually want to have children and you already doubled up on birth control*, *it might be very hard for you to actually have children in the future… Cause especially when you've been on birth control for so long… it would be really hard for you to get pregnant*.*”*

Issa, U.S., 19 years, HIV negative, African American/Black

#### Undue burden on women

Numerous participants noted that the contraception requirement was nonsensical for women who are not sexually active.

“*At least if you are getting an injection when you have a husband*, *it can be good*. *Because you will know that with the husband I have*, *it will make sense*. *But if you do not have a lover*, *you don’t have a husband and you should find yourself injecting*, *it is not good*. *How can one get pregnant…*?*”*

Chikondi, Malawi, 25 years, HIV positive, Black African

*“Well, if I don’t have a current sexual partner*, *then what would be the point of being on birth control? And if the birth control doesn’t have any bearings on the actual study, then why do I need it?”*

Robin, U.S., 37 years, HIV positive, African American/Black

Several women in the US challenged the requirement for two types of birth control, believing one should be sufficient, and felt that it placed an undue burden on women. Some of these participants cited the relative effectiveness of different forms of birth control, arguing the number of forms required should depend on if one is long-acting/highly efficacious or not.

*“(Two types of birth control) is excessive… (W)ith the long acting birth control you don't really need two forms … If it's not a long acting birth control then I think yes*, *maybe you need a backup …”*

Jasmine, U.S., 32 years, HIV negative, multi-racial

Another participant described what she believed was a more fair and rational approach, and also how she would circumvent this requirement to participate in a trial.

“*I think that they should say you have to use a form of birth control that gives you X amount of protection and then they should give you different options and say if you want to have an IUD that’s fine … If they say it needs to be ninety-eight percent effective and you want to use condoms and that’s only ninety percent*, *then they say you need to do something else and here are your options but I don’t think it should be you have to use (two forms)*. *That’s just ridiculous… I would just tell them what they want to hear*. *I mean I would*, *I’m sure a lot of people would do that… So yeah*, *so I would lie* …*”*

Wendy, U.S., 30 years, HIV negative, white

A few US women discussed that they are disinclined to take any medication, so that this requirement would create a barrier to their participation.

“*I like to do a lot of things… the natural way… I’m not really big into a lot of medication and… putting things into your body… So if you want me to take two types of birth control*, *I just think that’s a bit much*.*”*

Wanda, U.S., 33 years, HIV positive, African American/Black

#### Relationship concerns

Participants in Malawi raised concerns that the contraception requirement could potentially damage their marital relationships, especially if a woman was using birth control while her husband was away, causing suspicions of infidelity.

“*Your husband is away*, *and you are using contraceptives here*? *Your husband is at liberty to speculate you were doing wrong things*,*… ‘Why were you using contraceptive while I was away*?*’ … This destroys marriages…*.*”*

Sylvia, Malawi, HIV positive, 29 years old, Black African

Some women commented on the potential relationship problems that insisting on two forms of contraception could cause if the male partner has had a vasectomy, as it would imply the woman is being unfaithful.

“*So for someone whose husband had vasectomy*, *right*, *if the husband sees the contraceptive*, *he going to say you are doing it with other men*. *Yes*, *because your husband had vasectomy and I don’t have any other person to sleep with I don’t have anyone to impregnate me how am I supposed to use contraceptives*? *That is not appropriate*.*”*

Rhoda, Malawi, HIV negative, 33 years old, Black African

Another participant noted some men’s aversion to condoms, and that requiring two forms of birth control may lead to infidelity.

“*Some men don’t accept; they say I don’t want a condom… So let’s say he is your husband saying I don’t want a condom; it means you have given him a chance to go out with other women*.*”*

Faye, Malawi, 31 years, HIV positive, Black African

## Discussion

Our study is the first cross-national qualitative study to our knowledge of women’s views about contraception requirements for biomedical research participation. Among 140 women in the U.S and Malawi, a majority offered overall support for contraception requirements, while some women were opposed and others were unsure. Seven themes characterized the identified advantages and disadvantages of research-related contraception requirements generally, several of which were country-specific (see Table 3). Not only do our findings expand the conversation beyond considerations generally raised in debates about contraception requirements, but they highlight the ways that context shapes women’s experience–as well as the ethics–of requiring contraception in a clinical trial.

Specifically, while the proportion of participants in favor of or against contraception requirements were similar across the two countries, the reasons offered were not. Particularly notable is the contrast between Malawian and U.S. participants in their characterization of the burdens of contraception requirements. U.S. women tended to focus on *biomedical* concerns such as side effects and impact on fertility, and questioned whether requiring two forms of birth control was medically justified. In contrast, Malawian women focused on the *social* risks of birth control requirements, which could be profound: while the use of a contraceptive could lead to violations of trust in marital relations and suspicions of potential infidelity, it could also serve as a leverage point for women’s reproductive control.

Respondents in the U.S. and Malawi were more similar in the ways that they characterized the benefits of contraception requirements, though important differences remained. Many of the positives noted were related to contraception use in general, and not specific to a trial context. Women in both countries noted that contraception requirements could enhance reproductive control by reducing unintended pregnancy. If contraception was meaningfully accessible and its use supported outside the context of a study, it would seem that requiring contraception use by research participants would not in fact increase reproductive control. But contraceptives are not necessarily easily accessible for women in either setting, and their socio-cultural contexts may not support contraceptive use. For example, in the U.S., there may be financial constraints for women without any health insurance or with health insurance that does not cover all contraception types [29,30]. In the Malawian context, there may be cultural constraints such as a presumption that use of contraceptives implies infidelity in marriage, or structural constraints such as transport barriers, long wait-times, and limited contraception options. In both contexts, then, a research participation requirement that mandates and supplies contraception can be both useful to and welcomed by women who do not desire pregnancy. In the Malawi context, for example, contraception requirements for trial participation were seen as facilitating birth spacing, which has important health benefits for women and children alike.

To the extent that a research requirement for contraception is welcomed by women whose use of contraception is otherwise constrained, some may question whether a requirement for and provision of contraception could represent undue inducement for some participants. While contraceptives may be an ancillary benefit for those who desire them, undue inducement pertains only when an offer has a distorting impact on a research participant’s ability to rationally weigh costs and benefits of participation [31]. Nothing respondents in our study said indicate such a distorting influence; and none offered that access to contraception itself would change their mind in favor of participation. Further, given the unfortunate constraints on contraception use for some women outside of a research context, the reasons participants offered in favor of access are entirely rational.

Whether in favor of or against contraception requirements, our data highlight the importance of considering how socioeconomic and cultural context–gender, race, socioeconomic status, cultural considerations–shape women’s views about contraception. In recent years, discussions of ethics and reproduction have increasingly employed what is known as a “reproductive justice” framework. As opposed to a “reproductive rights” framework, which tends to focus on rights of access to contraception and abortion, the reproductive justice framework takes a broader view and considers the myriad issues that affect women’s reproductive lives. Centrally, the framework recognizes women’s interests in having (as well as not having) children and being able to raise them in a safe, healthy environment [32]. The concept of justice thus extends beyond ensuring legal access to means to prevent or discontinue pregnancy to ensuring fair access to resources, non-discriminatory policies, and institutional structures that enable women to maintain control over their bodies and lives, and would press questions not just about fair access to contraceptives but the ethics of requiring that women use them [33]. Critically, the reproductive justice framework also considers how gender, race, and socioeconomic status shape access to and experiences of reproductive health care, including within clinical research [34]. In keeping with the reproductive justice framework, the differential concerns of Malawian and U.S. women reflect the important impact that cultural context may have on women’s views about and experiences of contraception requirements in clinical studies. In particular, in Malawian contexts, where society has been described as largely patriarchal [35,36], contraception requirements may be seen as a way for women to work around gender power differentials when contraception is desired; or as a burden that may engender conflict in marital relationships. A reproductive justice framework would attend to both sets of concerns as well as the ways in which cultural norms around gender shape the experience of the requirements–both in terms of creating opportunities for empowerment through contraceptive access and introducing risk for women who desire study participation. As such, a reproductive justice framework would potentially entail a shift away from an emphasis on required use, and toward approaches that support women’s informed choices about use or non-use of contraceptives, as well as access to desired contraceptives in research contexts.

For the research community, contraception in clinical trials is motivated by various factors, often in combination. These include a desire to protect the fetus from research risks, reduce costs and product development timelines, protect from liability due to pregnancy exposures in clinical trials, and maintain the “statistical integrity” of studies that can be compromised by high pregnancy rates associated with study withdrawal [22,37]. Some women in both the U.S. and Malawi felt that researchers–by virtue of their authority as researchers–should be able to impose contraception requirements. However, many respondents’ reasons for favoring contraception requirements included the benefits of contraception generally, rather than being solely motivated by a desire to protect their potential offspring. Also, from our Malawi respondents we learned that contraception requirements entail burdens for women that may not have been previously appreciated. While a range of external considerations such as sponsor liability are likely to inform decisions about contraception requirements, our findings suggest at least four ways in which such requirements could be more responsive to and respectful of research participants.

First, contraception requirements should be sensitive to actual fetal risk assessments. In studies involving women, contraception requirements have tended to be presumed to be justified–and necessary–even where fetal risk is uncertain. For instance, women of childbearing age may be required to use highly effective methods of birth control and pregnancy tests when participating in early phase trials before non-clinical reproductive toxicology studies have been conducted [8]. But there are many circumstances where contraception is required in which the risk of harm to offspring is extremely low and well-characterized. As some of our U.S. participants indicated, whether contraception requirements are justified under these conditions is, at best, questionable. Given the burdens made vivid by our study, it may be more appropriate and respectful of women’s interests to require that researchers justify any requirement for contraception–and not only in terms of the likelihood of harm of early fetal exposure to a drug or biologic but also in terms of the likelihood of incident pregnancy. Factors to be considered should include sexual activity and orientation of participants, the potential burdens of contraceptives both in terms of biomedical and social risks, and the likelihood that women of childbearing age will use the intervention in clinical contexts and become pregnant.

Second, wherever possible, researchers should strongly consider providing access to contraception, even where pregnancy-associated risks of research are minimal and contraception requirements are unwarranted. There is increasing recognition that researchers have responsibilities to provide needed medical care that is beyond the narrow purview of their research (ancillary care) [38]. Access to contraception is widely recognized as important to women’s health and empowerment, and global disparities in such access is a topic of ongoing concern [39,40]. Given that many of the benefits that women reported from contraception requirements were actually benefits of having access to contraception that might otherwise be challenging to obtain, ensuring access to contraception as a form of ancillary care should be considered in research planning and implementation.

Third, when contraception requirements are deemed appropriate, they should be directed at optimizing effective pregnancy prevention rather than a blunt approach of mandating “two forms” of contraception absent clear evidence of improved protection. Interestingly, where prevention of pregnancy is a priority, “clinical guidelines and national data” have indicated that “using one effective method [of contraception] correctly and consistently” is the best way to avoid pregnancy in clinical settings, and that providing choice over modality increases use [41,42]. There is no reason to believe that the research context would be different. Additionally, individual factors, including if a woman exclusively has sex with women, is abstinent, or infertile should be explored and integrated into assessing the appropriateness of the requirements. Incorporating evidence-based contraceptive counseling that is responsive to women’s needs and life circumstances, and allowing a range of contraceptive options, may serve a dual purpose of respecting women’s autonomy as well as reducing unintended pregnancies in research contexts, while also potentially increasing trial eligibility, recruitment, and retention.

Finally, our findings may have implications for understanding the high rates of contraceptive failure in clinical studies [37,43,44]. Research on the psychology of contraceptive use indicates that women’s efforts to avoid pregnancy are based on a “benefit:burden ratio” and that contraceptive use depends on a range of evolving factors, including positive and negative views about becoming pregnant as well as perceptions of and experiences with specific contraception methods [38,45,46]. We are not aware of any studies on the psychology of contraceptive use where contraception was required in clinical research. One observational study cited misconceptions and incorrect use as contributing to unintended pregnancies in trials [47]. Non-adherence to study requirements for contraception may be high where motivations regarding pregnancy are at odds with study requirements, there is ambivalence about contraceptive methods or use, and social contexts may weigh heavily on decisions about pregnancy and contraception. In addition to addressing high rates of contraceptive failure, it may be prudent to be more deliberate and reasoned about when contraception should be required, and consider whether offering more contraceptive choices in the context of studies could lead to a more robust evidence base that meets the needs of women across their reproductive lives.

Several considerations inform interpretation and generalizability of our results. Our qualitative methodology was intended to identify and explore women’s opinions on contraception requirements in order to surface themes and concrete considerations that policy makers should be cognizant of when determining regulations and best practices. It was not intended to measure the prevalence of such views among women, or to assess women’s views about their appropriateness in specific study contexts with varying levels of potential risks to the fetus, both of which may further inform future policies. Our sample is not representative of all women at risk for or living with HIV in the U.S. and Malawi, or women of reproductive potential more generally. Our results cannot be generalized to the population of women who might be eligible for research participation.

Our study has several limitations. The sample for this study included pregnant women, for whom the contraception requirement would not be immediately applicable. How pregnancy may affect women’s views of contraception requirements while participating in research remains unclear. Additionally, a more representative sample of reproductive-aged women at risk for or living with HIV may include different proportions of women who are infertile, who do not desire pregnancy, or who exclusively have female sex partners, characteristics which are likely to influence views on clinical trial contraception requirements.

Future qualitative research with a complementary sample of women of reproductive age who are neither pregnant nor recently pregnant would be useful to explore if substantive variations in views related to these considerations exist. Additionally, research using quantitative methodologies would be beneficial to more clearly delineate the prevalence of and potential associations within these views. Further, while this particular set of questions referenced research generally and did not specify a particular health condition, respondents were primed to respond to HIV-related research with interview questions that preceded this one; their answers may have been influenced by these prior references. We did not ask women to consider specific types of contraceptives, nor did we ask them to reflect on how level of or uncertainty about fetal risk, or other potential justifications for contraception requirements, would affect their views. All would be important topics of future study.

Requiring a woman to use contraception in any circumstance is ethically complicated. Yet in the context of biomedical research with women of childbearing age, contraception requirements have often been viewed as necessary to the ethical conduct of research–or an allowable option for minimizing R&D timelines and costs by delaying developmental toxicity studies. Our study highlights the ways in which contraception in the context of research may be viewed as either valuable or burdensome to women, depending on social context, and underscores the ethical complexity of contraception requirements in clinical research. These findings highlight considerations important to developing ethical guidance for the provision or requirement of contraceptives in the context of clinical trials, and to robustly orienting such trials around the interests and lives of the women who will participate in them.
