# Correction: Women's views about contraception requirements for biomedical research participation

**DOI:** 10.1371/journal.pone.0218424

**Published:** 2019-06-12

**Authors:** The PLOS ONE Staff

There are a number of errors in the formatting of Table 3. The authors have provided a corrected Table 3 below as Supporting Information file S1 File.

## Supporting information

S1 File Corrected Table 3. Themes emergent across participants’ assessments of advantages and disadvantages of contraception requirements.
(PDF)
