# Supplementary material for: Correction: Women's views about contraception requirements for biomedical research participation
Source: PLoS One. 2019 Jun 12;14(6):e0218424. doi: 10.1371/journal.pone.0218424 (PMC6561573; doi:10.1371/journal.pone.0218424)
Supplement: S1 File — Themes emergent across participants’ assessments of advantages and disadvantages of contraception requirements. (PDF) [file pone.0218424.s001.pdf]

Table 3. Themes Emergent Across Participants' Assessments of Advantages and Disadvantages of Contraception Requirements

Domain	Advantages of a requirement	Country	Disadvantages of a requirement	Country
Reproductive control	Access to birth control/prevention of unintended pregnancies	U.S. & Malawi		
	Pregnancy prevention enhanced by 2 types of birth control	U.S.	Reduced control of pregnancy timing	U.S. & Malawi
	Male partners respect requirement so women get access to benefits	Malawi		
Health effects	Protection from sexually transmitted diseases (with condom use).	U.S.	Side effects of birth control	U.S. & Malawi
	Improved birth spacing	Malawi	Side effects of birth control magnified with two forms of contraception	U.S.
Preventing fetal harm	Fetal protection from unknown risks of study drug	U.S.		
	Requirement is appropriate when potential risk for fetal harm is present*	U.S.	Requirement is not appropriate when potential risk for fetal harm is absent*	U.S.
	Liability protection for researchers	U.S.		
Burden on women			Birth control is unnecessary if not sexually active	U.S. & Malawi
	Low incremental burden on women already using one form	U.S.	Using two types of birth control is burdensome.<br>- One long-acting/highly efficacious type should be sufficient	U.S.
			Desire to minimize medication use and be "natural"	U.S.
Deferral to authority	Researchers have the right to establish the rules	U.S. & Malawi	--	
Autonomy to enroll and choice of birth control method	Women can make informed choice about participation in the study	U.S.	--	
	Participants get to select which form(s) of birth control they use	U.S. & Malawi		
Relationship concerns	--		Partner may suspect infidelity if he is away and woman is using contraception, or he has had vasectomy	Malawi
			Man may find an alternative sexual partner to avoid using condoms	Malawi

*Participants indicated appropriateness of requirement is dependent on potential of risk for fetal harm
